# The Implementation of Advanced Practice Nursing in Primary Health Care: A Comparative Qualitative Study of Enablers and Barriers

**DOI:** 10.1111/jan.70172

**Published:** 2025-08-27

**Authors:** Daria Bula, Cassiano Mendes Franco, Lígia Giovanella, Beatriz Rosana Gonçalves de Oliveira Toso, Marcus Heumann, Kerstin Hämel

**Affiliations:** ^1^ Department of Health Services Research and Nursing Science, School of Public Health Bielefeld University Bielefeld Germany; ^2^ Faculty of Medicine Federal University of Rio de Janeiro Rio de Janeiro Brazil; ^3^ National School of Public Health Sergio Arouca, Oswaldo Cruz Foundation Rio de Janeiro Brazil; ^4^ Western Paraná State University Cascavel Brazil; ^5^ Department of Nursing Science, Faculty of Social Sciences University of Vienna Vienna Austria

**Keywords:** advanced practice, chronic illness, focus groups, nurse practitioners, nurse prescribing, nurse roles, nurse–physician relationships, policy, primary care

## Abstract

**Aim:**

To explore the enablers of and barriers to implementing advanced practice nursing in primary health care in Germany and Brazil.

**Design:**

A qualitative cross‐country comparative study.

**Methods:**

Nine focus groups were conducted: 4 in Brazil and 5 in Germany with 48 participants (23 primary health care policy stakeholders and 25 nurses practicing in primary health care and general practitioners) between May 2022 and June 2023. The data were analysed by content analysis using a deductive–inductive approach.

**Results:**

Our findings reveal a need for clarity around the concept, specific roles and responsibilities of advanced practice nurses in primary health care. Although there is still no regulation in place for practising advanced practice nursing in either country, clear drivers can be observed, with Germany strengthening community health nursing and Brazil following clinical protocols in nursing practice. Dialogue among stakeholders—at both the policy and practitioner levels—is essential to bridge communication gaps. Additionally, involving patients in the implementation process is crucial for the holistic integration of advanced nursing roles.

**Conclusions:**

Political, organisational and financial barriers persist, such as the need to establish both legal foundations and regulatory frameworks, enhance political participation within the nursing profession, and involve stakeholders in dialogue and consensus‐building efforts. Giving advanced practice nursing a higher priority on political and research agendas—with policy adjustments and input from practitioners—can help integrate advanced practice nursing into primary health care.

**Implications for the Profession and/or Patient Care:**

Our findings highlight that actively involving nursing as an equal partner in political discourse is seen by stakeholders as crucial to drive the implementation process forward sustainably.

**Impact:**

This study addresses the lack of data on the enablers and barriers to implementing advanced practice nursing in primary health care in Germany and Brazil. It underscores the need for clearer definitions of advanced practice nursing in primary health care, as well as sufficient regulation and funding. Dialogue is essential to bridge gaps and foster mutual understanding. The findings support future practice development and research, especially in countries that have introduced advanced nursing practice roles in primary health care.

**Reporting Method:**

The COnsolidated criteria for REporting Qualitative research (COREQ).

**Patient or Public Contribution:**

No involvement of patient and public contribution.

**What Does This Paper Contribute to the Wider Global Clinical Community?:**

Our study highlights the growing adoption of expanded nursing responsibilities even in countries that have not yet formally implemented advanced practice nursing roles.

**Summary**

- What already is known
  ○ Implementing advanced practice nursing in primary health care is a multifaceted and complex process.
  ○ The level of advanced practice nursing implementation varies significantly across countries.
  ○ Although various barriers and enablers have been identified, context‐specific knowledge—especially for Germany and Brazil—remains limited, yet essential to guide implementation.
- What this paper adds
  ○ Stakeholders and practitioners of primary health care in both Germany and Brazil emphasise the need for conceptual clarity around advanced practice nursing roles in primary health care.
  ○ Dialogue between the political and practical stakeholders of primary health care is perceived as essential to bridging communication gaps during the implementation process.
  ○ A lack of legal regulation and insufficient funding are perceived as major barriers in both countries.
- Implications for practice and policy
  ○ Nurses should play an active role in shaping policy and regulatory frameworks related to advanced practice roles.
  ○ Developing country‐specific legal frameworks to define the scope of advanced nursing practice is vital for establishing a shared understanding and facilitating implementation.
  ○ Elevating implementation of advanced practice nursing to the national political agenda is critical for achieving sustainable change.

## 1 Introduction

The inclusion of advanced practice nursing (APN) in primary health care (PHC) is seen as a key strategy for addressing complex health needs (Brownwood and Lafortune 2024). According to the International Council of Nurses (ICN), advanced practice nurses (APNs) possess advanced clinical competencies and complex decision‐making skills that require at least a master's level of education (ICN 2020). While the classification of APN roles varies internationally, APN is commonly defined by a set of core competencies spanning four key pillars: clinical practice, leadership, management and education (ICN 2020). Reflecting this multidimensional profile, the World Health Organization (WHO) underscores the role of APNs in improving health care access and quality and in strengthening the nursing workforce. Therefore, it advocates their broader global adoption (WHO 2020).

The implementation of APN is a multifaceted process that requires a systematic approach to clearly delineate APNs' roles and responsibilities (Andregård and Jangland 2015; Bryant‐Lukosius and Dicenso 2004). Research indicates that adopting APN roles involves both challenges and enablers, which are shaped by country‐specific contexts (Torrens et al. 2020). It is therefore crucial to investigate the specific barriers and enablers in each country, as these factors significantly influence either the success or failure of APN implementation. This is particularly pertinent for countries such as Germany and Brazil, where APN is still at an early stage of development (Stemmer et al. 2023; Miranda Neto et al. 2018).

## 2 Background

APNs possess different skills that help them provide clinical practice, leadership, management and education within different health care settings. APN development is shaped by the fundamental level of nursing practice and education present in a country. The implementation of APN also depends on both the professional status of nursing and the country's capacity to introduce new nursing roles (Schober et al. 2016). In addition, depending on the country‐specific PHC context, APNs are often integrated into multi‐professional teams that include various health professions such as medicine, midwifery, pharmacy and allied health (Delamaire and Lafortune 2010; ICN 2020).

The participatory, evidence‐based, patient‐centered process for APN role development, implementation and evaluation (PEPPA) framework is a recognised tool for APN role establishment (Unsworth et al. 2022). It consists of a nine‐step model that explores the full range of the role implementation and evaluation process (see Table 1).

**TABLE 1:** Nine steps of the PEPPA framework (Bryant‐Lukosius and Dicenso 2004), own illustration.

Steps	Components of the steps
1. Define patient population and describe current model of care	Identify the target group of patients or population that will benefit from the role
2. Identify stakeholders and recruit participants	Analyse the existing health care delivery approach, including gaps and limitations
3. Determine the need for a new model of care	Propose an improved care model that integrates advanced practice nursing
4. Identify priority problems and goals to improve the model of care	Define the main challenges to be addressed and the intended outcomes
5. Define new model of care and APN role	Involve patients, health care professionals and policymakers to align objectives and expectations
6. Plan implementation strategies	Develop concrete strategies and actions for implementing the APN role
7. Initiate APN role implementation plan	Put the APN role into practice within the health care system
8. Evaluate APN role and new model of care	Assess the effectiveness and impact of the role and the new model on patient and system outcomes
9. Long‐term monitoring APN role and model of care	Ensure long‐term success through ongoing evaluation of performance against initial priorities

The approach outlined by the PEPPA framework highlights that careful planning is required for the successful implementation of APN roles. However, the adoption of these roles varies across health systems. In practice, a lack of cohesion is often observed; for example, in Brazil, some APN tasks are carried out by nurses without specific role delineation, whereas in Germany, APN master's programs are offered without a legal framework or defined scope of practice (Bula et al. 2024). Andregård and Jangland (2015) described the often unsystematic and bumpy implementation process as a ‘tortuous journey’ (3).

According to a scoping review by Torrens et al. (2020), the key facilitators for implementing APN roles in PHC include supportive legislation, well‐defined role parameters, recognition within the health system, the integration of APNs into interprofessional collaboration and acceptance by other health professionals. In contrast, barriers are predominantly related to insufficient regulatory frameworks, limited funding, the health system in general and unclear role delineation. Additional challenges include resistance and limited acceptance among physicians, as well as a general lack of understanding of the APN role (Torrens et al. 2020).

### 2.1 Implementing APN in PHC in Germany and Brazil: An Overview of the Context of the Study

Germany's health system is physician‐centered, with PHC being largely provided by general practitioners (GPs), who are supported by medical assistants. PHC in Germany also comprises other health professionals, including nurses working in home care; however, such care must be prescribed by GPs (Bula et al. 2024). Expanded roles for nurses in PHC and APNs have been piloted in several small‐scale model projects (Stemmer et al. 2023).

In contrast, in Brazil, in accordance with the Family Health Strategy (FHS) of the Unified Health System (SUS), PHC is provided mainly by family health teams involving 1 physician, 1 nurse, 1–2 nursing technicians and 4–6 community health workers (Franco et al. 2025). Nurses in Brazil play a much more autonomous and integral role in PHC than do those in Germany, including the handling of specific tasks related to health promotion, disease prevention, and treatment (Franco et al. 2025). They can diagnose and prescribe for certain conditions following guidelines and often serve as the leaders of family health teams and clinics (Magnago and Pierantoni 2021).

In Germany, most fully educated nurses achieve their qualifications after completing three years of vocational training in nonacademic nursing schools rather than through academic routes. With the 2020 Nursing Professions Act, the academisation of nursing through a foundational bachelor's programme became legally regulated (Bula et al. 2024). In addition, in Germany, it is possible to pursue APN qualifications at both the bachelor's and master's levels, although the bachelor's qualification pathways differ from the ICN definition, which specifies a master's degree as the expected standard (Bula et al. 2024; ICN 2020). In 2025, the federal government announced a willingness to establish an APN act for regulating this nursing role in Germany (Deutscher Bundestag 2025). Currently, APN roles do not exist in PHC; however, the introduction of a community health nurse (CHN) role, which is qualified at the master's level, is under active consideration (Brownwood and Lafortune 2024).

In Brazil, all nurses obtain a bachelor's degree in nursing, which includes training in patient care, public health and disease prevention. Postgraduate courses in family health or public health further enhance the skills of PHC nurses, ensuring that they can effectively contribute to Brazil's FHS (Bula et al. 2024). Since 2015, representatives from the Federal Nursing Council (COFEN) and the Brazilian Nursing Association (ABEn) have been collaborating with the Brazilian Ministry of Health and the Pan American Health Organization to explore strategies for implementing APN in Brazil (Minosso and Toso 2021; Miranda Neto et al. 2018).

Comparing countries with distinct PHC models, nursing roles, and nursing education systems—such as Brazil and Germany—offers valuable insights into the similarities and differences in barriers and facilitators. This nuanced approach provides new insights and can aid policymakers and stakeholders in developing strategies, thus addressing the unique challenges and opportunities in each setting while also enhancing our understanding of common and unique factors that impact APN integration.

In this light, we selected Germany and Brazil as contrasting cases for our comparative study, as they offer unique yet complementary insights into APN implementation. Germany is only beginning to academically train nurses but has already launched targeted APN model projects. In contrast, Brazil presents a long‐standing model of academically trained nurses with a strong role in PHC but without a defined APN framework. This contrast allows for an in‐depth examination of how different health system structures, levels of professionalisation, and degrees of institutional readiness influence the pathways of implementing APN roles in PHC.

## 3 The Study

This study is part of the research project ‘Strengthening Advanced Nursing Practice and Collaboration in Primary Health Care: Brazil and Germany’, whose overarching goal is to explore the possibilities and obstacles associated with APN implementation and the development of interprofessional collaboration in PHC in Germany and Brazil. This article aims to carry out a comparative analysis of enablers of and barriers to APN implementation in PHC in both countries; it does so by investigating the following research question: *What are the enablers of and barriers to implementing APN in PHC in Germany and Brazil, in terms of both policies and practices?*

The data collection and analysis of this study were informed by the PEPPA framework, highlighting that both practice‐level and policy‐level factors play crucial roles in APN implementation in PHC. In addition, the framework helped structure our focus on relevant implementation aspects and guided reflections on potential strategies for advancing APN roles in a context‐sensitive and evidence‐informed manner.

Further findings from this research project's empirical analysis—specifically on shared care and collaboration between PHC nurses and physicians—have been published in separate articles (Franco et al. 2025; Felix et al. 2025).

## 4 Methods

### 4.1 Design

Employing a qualitative research design, we delved into the perspectives of PHC stakeholders and practitioners and compared their views on ongoing and future APN implementation in Germany and Brazil. Data were collected by conducting focus groups in both countries.

Given the exploratory nature of our research questions and the cross‐country study design, we selected qualitative content analysis (QCA), a method with a long‐standing tradition in nursing research. QCA provides a systematic yet flexible way to reduce and interpret qualitative data by organising large text volumes into meaningful categories (Elo and Kyngäs 2008). It also allows for the integration of predefined models—in the case of our study, the PEPPA framework (Bryant‐Lukosius and Dicenso 2004)—while remaining open to emerging, data‐driven insights, thus combining deductive and inductive reasoning in a way that supports both theoretical alignment and sensitivity to context‐specific patterns (Elo and Kyngäs 2008; Schreier 2012).

### 4.2 Selection of Study Participants

The focus groups were conducted at the policy (1) and practitioner (2) levels. At the policy level, we involved stakeholders in PHC policy‐making. At the practitioner level, we involved physicians and nurses practising within PHC. For participant recruitment, we employed purposive sampling to select stakeholders based on our knowledge of the policy field. We included leaders representing key organisations such as health councils, funding bodies, and professional associations at both the national and regional levels in Germany and Brazil. For practitioners, a convenience sample was chosen based on expert recommendations and by contacting gatekeepers. The research team ensured suitable and comparable selection for both countries, including an equal mix of physicians and nurses. Participating GPs and nurses were required to have at least one year of experience in the FHS (Brazil) or in GP practices and/or home care services (Germany), with all nurses holding at least a bachelor's degree.

In Germany, recruitment involved participants from nine of the sixteen states; we targeted all PHC nurses with expanded roles in Germany, as only a few PHC projects exist across the country. We identified these nurses based on desk research and our knowledge of the field. In Brazil, we targeted national stakeholders, practitioners and regional stakeholders from Paraná and Rio de Janeiro for recruitment because both states provide robust PHC training programmes and effective PHC coordination; the location of the Brazilian research team in these states facilitated access to the field.

### 4.3 Study Setting and Recruitment

The focus group guidelines incorporated the principles of the PEPPA framework (Bryant‐Lukosius and Dicenso 2004), including five key subjects (see Table 2) (Franco et al. 2025).

**TABLE 2:** Overview of the focus group guidelines (Franco et al. 2025).

Theme	Aspects
Establishing the focus of the session: Introduction to the overall topic by the moderator, definition of APN according to the International Council of Nurses, illustration of the development of APN in different countries	
1. Understanding of and experiences with APN	Points of contact and experiences of the participants with APN
2. Collaboration between physicians and nurses in PHC	Current collaboration, needs for improvement, opinions regarding increasing the autonomy of nurses in patient care
3. Strengths, possibilities, and obstacles associated with the implementation of APN	Current tasks of nurses, facilitators and obstacles to the implementation of APN in PHC
4. Role of institutions in the implementation of APN	Management responsibilities, role of professional associations
5. Visions for the future/conclusions	Visions of collaboration and the implementation of APN in 10 years

Insights and feedback from a pretest were used to make minor adjustments, refining the guideline until consensus in the research team was reached.

Participants were contacted via either email or telephone and provided with written study information. From May 2022 to June 2023, nine focus groups were conducted with a total of 48 participants. In each country, we hosted two focus groups involving practitioners, one comprising federal stakeholders, and three focus groups (1 in Brazil, 2 in Germany) with regional stakeholders at the state level. To maintain the participants' anonymity, the codes attributed were nursing stakeholder (SHN), physician stakeholder (SHP) and other stakeholder (SHO). In the practitioner focus groups, the acronym RN was assigned to nurses, while that of GP was assigned to physicians, followed by the number of the interview in consecutive order.

### 4.4 Data Collection

The focus groups were led by trained moderators from our research team in either German (Germany) (KH, DB, MH) or Portuguese (Brazil) (BT and other research members). Owing to logistical constraints related to participant dispersion, eight focus groups were conducted online via Zoom Video Communications, whereas one focus group with practitioners in Brazil was held in person. The sessions lasted between 2 and 3.5 h each. All interactions were videorecorded, transcribed and subsequently translated into English. Portuguese and German speakers of the author team who are proficient in English supervised the transcription and translation processes to ensure the accuracy of the data.

### 4.5 Data Analysis

The transcripts were anonymised and analysed following QCA according to Kuckartz and Rädiker (2023) via a deductive‐inductive approach. Coding was conducted in MAXQDA 2022 software. The general workflow comprised five steps: reading the data intensively, building the coding frame, coding the data, analysing the coded data and interpreting the results (Kuckartz and Rädiker 2023). Throughout the process, the research question remained the central focus.

An intensive reading of the data provided the foundation for forming categories and the multicycle coding process. In the first step, categories were formed via a deductive approach, guided by the focus group guidelines with consideration of the PEPPA framework and the research question. In the second step, the first author applied these categories to the material. She then inductively coded both the barriers and enablers for each category. In the author team, we continuously discussed the identified barriers and enablers. The inductive coding also led to a further development of the previously deductively applied main categories and readjustments. This iterative combination of deductive and inductive analysis allowed us to remain theoretically guided while staying open to new, data‐driven insights—a key strength of QCA (Schreier 2012). Finally, the category structure was reorganised through three main categories of identified enablers of and barriers to APN implementation (see Table 4), and interpretations were revised and further developed within the whole team of coauthors.

### 4.6 Ethical Considerations

The research program was approved by the ethics committees of Bielefeld University (reference number: 2022‐87) and Western Paraná State University (UNIOESTE) (reference number: 5.349.117). All participants were provided with participant information in their respective languages and gave their written consent to participate in the focus groups. An agreement for the cooperative responsibility of processing data between the German and Brazilian research institution teams as well as individual written agreements facilitated the assurance of data confidentiality.

### 4.7 Rigour and Reflexivity

We conducted the study in accordance with the four principles of rigour (Lincoln and Guba 1985). Credibility was supported through the open design of the focus group discussions, in which participants were explicitly encouraged to freely express their experiences. Although coding was primarily conducted by the first author, regular reflexive discussions within the multidisciplinary and international coauthor team were held. This also contributed to confirmability, which was further supported through the joint interpretation of the German and Brazilian data. Dependability was ensured through a deductive‐inductive coding procedure following Kuckartz and Rädiker (2023). Finally, transferability was supported by detailed contextual information on both health care systems and a precise sample description.

## 5 Findings

### 5.1 Characteristics of the Study Participants

Table 3 presents the study participants' characteristics; there were 48 participants in total, with 19 from Germany and 29 from Brazil, and a total of 23 stakeholders and 25 practitioner.

**TABLE 3:** Characteristics of the study participants from the focus groups.

Participants	Germany	Brazil	Germany and Brazil
Sex			
Total	19	29^a^ (30)	48
Male	6	9	15
Female	13	20	33
Stakeholders total	10	13	23
Profession			
Nurses	5	7	12
Physicians	3	2	5
Other professionals	2	4	6
Practitioners total	9	16	25
Profession			
Nurses	6	9	15
Physicians	3	7	10

^a^ One participant was present only at the beginning and did not contribute to the focus group.

**TABLE 4:** Overview of categories, enablers and barriers identified.

Category (description)	Barriers	Enablers
*Understanding of APN in PHC* (This category summarises statements from participants on how they perceive the term ‘APN’, what they associate with it and where there are still difficulties in understanding it)	There is no clear definition of APN in the health system and PHC model of the country (Ger/Bra) Physicians hold power within PHC, which often leaves nurses in a weaker position to develop their roles (Ger/Bra) APN could be seen as a ‘stopgap’ solution to address the shortage of physicians in PHC (Ger/Bra)	Advanced clinical nursing tasks have been successfully implemented within the health system (Bra) APN model projects help to provide a better understanding of tasks and chances for care quality (Ger) Implementing APN roles aligns to the PHC concept and addresses population health needs (Ger/Bra)
*APN on the political agenda* (This category summarises statements from participants that represent enabling and hindering aspects at the political level)	Advanced clinical tasks of nurses are legally forbidden, no legal liability for nurses (Ger) Protocols for nurses are in part insufficient and contradictory (Bra) The underfunding of the health system hinders the adequate renumeration of APNs (Bra) Fragmentation of medical and nursing care in PHC (Ger) Nurses in practice are partly in opposition to ‘innovations’ such as APN (Ger/Bra)	Political initiatives favour the development of CHN (Ger) Various protocols that enable an expanded scope of practice for nurses in PHC are already implemented in practice and politically supported (Bra)
*‘Have to sit down at the table’: Dialogue between stakeholders* (This category summarises statements made by the participants that highlight the importance of the necessary dialogue at stakeholder level, practitioner level and social level)	Lack of mutual exchange between the different stakeholders, particularly nurses and physicians, at policy and practitioners' level Patients typically prefer consulting physicians over nurses, often due to perceptions of physicians having higher levels of expertise (Ger/Bra) Nurses are absent in political discussions and often lack political representation due to the incomplete implementation of nursing boards (Ger) Population needs are not at the center of discussions on the development of APN (Ger/Bra)	Organization and active political participation of the nursing profession in health policy debates and processes (Bra) Positive attitudes of some physicians regarding the introduction of APN in dialogue with nurses (Ger/Bra)

Abbreviations: Bra = Brazil; Ger = Germany.

#### 5.1.1 Understanding APN in PHC

Participants from both countries acquired knowledge about APN primarily from academic settings, conferences and international literature rather than through everyday practice. They tended to have a more abstract rather than a practical understanding. Particularly in Germany, nurses at both practical and political levels reported engaging with the concept of APN through their familiarity with nursing education programs, recognising APN as a distinct pathway within these programs. Some German participants, even among physicians, reported having a practical understanding due to having participated in or discussed projects that trialled APNs in PHC or hospitals. However, some of the representatives of physician organisations and nurses' representatives at the practical level in Brazil confessed that ‘the term “advanced practices” is new to me’ (Bra, SHP).

The participants recognised that ‘APN consists of nurses who have different characteristics, such as having a broader or expanded scope of practice’ (Bra, SHN). Despite this more general assumption, the participants related APN to nurses who are able to perform advanced clinical tasks that are traditionally reserved for physicians. Regarding international standards, no one mentioned research or leadership and education for other nurses.

In Brazil, enhanced clinical practices, such as prescribing certain antibiotics, are already performed by general nurses in PHC if protocols are issued at the state and municipal levels. Some municipalities provide related training, thus creating different levels of expanded nursing practice in PHC across the country.

For Germany, the participants often referred to expanded nursing practices within model projects, in which tasks that were formerly restricted to physicians were successfully carried out by nurses. Typical extended tasks in these projects were reported to include performing ECGs, taking blood samples, controlling wounds and providing medication reviews. However, in Germany, both physicians and nurses at the policy and practitioner levels rejected the idea of granting nurses the authority to prescribe medications.

On the basis of our participants' ideas about APN‐associated tasks, it is considered common for Brazilian nurses to already perform tasks that are considered advanced in Germany; i.e., blood sampling, ECGs and medication reviews are routine tasks for general nurses. PHC nurses in Brazil practise with a fair degree of autonomy in many situations, particularly in the treatment of infectious diseases and in the field of child and maternal care. Nurses at both the policy and practice levels described numerous responsibilities in PHC nursing in Brazil; thus, they voiced being afraid that APN implementation would overload them with even more tasks.

While the participants provided ideas about what the APN concept entails and even made connections to what nurses already perform in practice, they were often elusive when attempting to concretize possible APN roles within PHC in their countries. This elusiveness was problematized by the nurses reporting that the term APN itself has not been sufficiently established in practice; APN ‘(…) is not discussed on a daily basis among colleagues (…) The impression I have is that my colleagues (…) have no information about it’ (Bra, RN). Furthermore, the Brazilian participants, in light of the already established broad scope of practice of nurses in PHC, indicated that the understanding of APN needs to be promoted in practice. They criticised that ‘this discussion is not being carried out effectively with nurses who work in PHC’ (Bra, SHN), highlighting the relevance of the conditions of both practice and the work environment for providing nurses room to manoeuvre in practice. In particular, German participants reported that APN is already in part implemented in some hospital environments; however, its translation to the unique context of PHC is less developed.

The participants criticised the absence of a clear delineation of tasks and the clarification of core competences associated with APN. They expressed concerns about the need to differentiate between the roles of APNs and those of general nurses. Given these issues, they questioned the feasibility of integrating APN into PHC.

Furthermore, the participants were bothered by the lack of differentiation in the task profiles of APNs from those of other health professionals, particularly physicians. In the case of Germany, participants stated that APN development should be discussed together with the development of enhanced tasks of medical assistants, as well as physician assistants educated at the bachelor's or even master's level. Some Brazilian nurses noted that they constitute ‘(…) *a profession that is still in search of its identity*’ (B1, Bra, SHN), indicating that the lack of definition of APN transcends the position of nursing itself in society.

Experts in both countries reported scepticism among nursing staff, particularly regarding the motives behind promoting APNs. They expressed doubts about whether the push for APNs originated from a genuine desire to empower the nursing profession or from workforce constraints, as has been experienced in the past. According to the participants' statements, APNs could represent a degradation of nursing if they are seen merely as substitutes for lacking professionals, particularly physicians.

Ah, there is no nutritionist, so the nurse has to do it; there is no social worker, so the nurse has to do it; there is no psychologist, so the nurse has to do it (…) This is an overload of work! (Bra, SHN)

On the other hand, given that nursing is generally regarded as being hierarchically lower than other health professions, the participants perceived the introduction of APNs as a general enhancement of the profession's reputation and an ‘immense potential to advance in valuing the nurse’ (B6, Bra, SHN). Consequently, there is an opportunity to strengthen work on ‘equal footing’ (E5, Ger, SHP) with the medical profession.

Despite the lack of ‘clarity*’* (Ger, SHN) of the term ‘APN’, participants from both countries engaged in discussions regarding its potential implications for PHC. The participants reported seeing an advantage for accessing the population and meeting the ‘health needs of the population’ (Bra, SHN), as APN strengthens patients' perspectives more. In addition, they recognised the benefit of bolstering regions that lack an adequate number of GPs. However, both nurses and physicians opposed the idea that APNs could serve as alternatives to physicians. In this context, APN should not be based on the ‘biomedical model*’* (Bra, GP). Instead, from the perspective of nursing, there is an opportunity for more patient‐centered care, with a holistic view, which positively affects population care by also considering ‘health promotion and prevention’ (I2, Ger, SHN).

One side of me is very happy about an increase in the scope of nursing, mainly because we know our population, and we know how much they need access, and the increase in the scope of nursing practice also means increasing health access and health promotion. (Bra, GP)

Specifically, in Germany, physician representatives at the policy level reported viewing the introduction of APNs as an opportunity to strengthen nursing in PHC overall, as PHC is still often considered ‘the appendix of hospital care’ (Ger, SHP).

#### 5.1.2 APN on the Political Agenda

From the point of view of our participants, one main factor related to the described elusiveness of APN is the current lack of laws that regulate APN in general and PHC specifically in both countries. Nurses' representatives from both countries highlighted the necessity of clearly differentiating APN roles from other nursing roles, such as general nursing, from the education system up to the jurisdictional level to prevent ambiguity.

Both practitioners and stakeholders emphasised the importance of regulatory oversight in shaping a clear and standardised legal framework for APN roles in PHC. The aim would be to ensure that expanded nursing activities are formally recognised and defined regarding the scope of practice, level of autonomy and legal responsibilities.

This need was perceived as being even more urgent in Brazil, as nurses are already involved in extended tasks. According to the Brazilian participants, such a framework could help reduce the uncertainty among practicing nurses, who sometimes find themselves in situations that lack adequate ‘legal support’ (Bra, SHN). From the German perspective, the establishment of a clear legal framework was reported to be particularly relevant, especially concerning liability law, which was reportedly often debated among the participating physicians.

If nurses determine the need for care in its assessment and have the possibility of prescribing nursing treatment (…) then they must ultimately be responsible for this area. (Ger, SHP)

Additionally, with respect to concerns about legal liability, the participants reported that nursing staff often grapple with a lack of self‐confidence.

They don't trust themselves to do it [expanded tasks]. They still have a lack of self‐confidence. (Ger, SHN)

In this context, especially in Germany, there is also a partially perceived ‘pronounced fear in nursing to make decisions themselves’ (Ger, GP) and take over responsibility, as described by the participants at both the policy‐making and practical levels. The participants reported that this attitude is counterproductive and prevents the nursing profession from becoming more attractive.

In Brazil, political advances to strengthen nurses' clinical responsibility can be observed, as there are regulations at the federal level that allow the establishment of extended nursing practices. However, the implementation of protocols varies at the state and municipal levels across the country. This heterogeneity creates inequality among nurses, with nurses in some regions performing advanced tasks that nurses in other regions are not allowed to perform, despite having the same educational background.

So, the practice of a nurse in Rio de Janeiro is different from the practice of a nurse in Acre. (Bra, SHN)

Similarly, German participants frequently addressed the overarching issue that the existing legal framework fails to accommodate expanded nursing practices. Moreover, such expansion is contested by key stakeholders of the German health system, including statutory health insurance funds and associations of statutory health insurance physicians, who are critical about its suitability for the German health system, as physicians are seen to oversee PHC. However, the participants reported observing a partial ‘willingness’ (Ger, RN) within the political sphere to explore the definition of APN within PHC, particularly within model projects.

That is also a form of political support for such projects as CHN and APN, which will play a big role. There's a great willingness right now to develop those kinds of projects. (Ger, GP)

Participants from Germany marked the implementation of CHN/APN master programs and corresponding model projects in PHC as promising approaches. However, both nurses and physicians reported doubting the pace of the wider implementation and stabilisation of these approaches. They criticised that model projects are often ‘unfortunately (…) terminated’ (G1, Ger, GP).

Additionally, experts reported that in model projects, even when physicians support the development of APNs, current barriers in the legal framework prevent effective implementation.

While the PHC center has employed (…) some nurses to engage in CHN, the problem is still that they cannot work independently in the way that would make sense. (…) there is still a dominance of the GP, even if these doctors do not want it that way. Legally, it is still the case that everything is still dominated by general practitioners. (Ger, SHP)

Participants from both countries highlighted funding feasibility as a crucial factor for APN implementation, repeatedly asking, ‘How do we finance this?’ (Ger, SHO). They stressed that regulatory issues include salary concerns, noting that increased autonomy and responsibility for nurses should be matched with fair remuneration. In addition, they noted a lack of funding opportunities to support APN roles in PHC. For Germany, this issue was attributed primarily to the strong fragmentation of the German health care system and the complex remuneration regulations, which are directed mainly toward physicians who are not inclined to allocate funds to APN. In Brazil, participants attributed the difficulty of providing funding for APN implementation to the general underfunding of the health care system.

#### 5.1.3 ‘Have to Sit Down at the Same Table’: Dialogue Between Stakeholders

The insights drawn from the focus groups underscore the importance of *‘dialogue’* (Bra, SHN) between the stakeholders of PHC at both the political and practical levels with regard to integrating APN roles into PHC. The participants observed a general ‘lack of conversation’ (Bra, SHO) between physicians and nurses. In addition to a political strategy, participants believed that promoting such dialogue could lead to a common understanding of APN. Furthermore, they emphasised the need for such dialogue at the societal level, with particular attention being given to keeping the population in mind when shaping APN.

At the policy level, the participants recognised that negotiation between the medical and nursing professions is essential for introducing APNs; however, this need is challenged by physicians' predominance in both countries. Thus, negotiations were framed as a critical step to create well‐defined competencies and avoid professional overlap.

In Germany, participants found it important that the ‘*main players, [who] are the KVs [Kassenärztliche Vereinigungen—*associations of statutory health insurance physicians*] and the insurance funds (…) would actually have to sit together and find a concept; otherwise, it would be difficult to apply*’. (Ger, GP)

Moreover, it was emphasised by Brazilian participants in the practitioners focus groups that PHC managers in municipalities should also be involved in this dialogue since they play a key role in shaping PHC in the SUS: ‘the [municipal health sector] management needs to understand and realize the importance of it [APN]’ (Bra, RN).

In Brazil, concerning institutional roles, nursing representatives emphasised the importance of fostering dialogue and collaboration not only among various other stakeholders but also among their own representatives in Brazilian nursing associations and nursing councils. For them, it is important to build a consensus to avoid disharmony and hierarchical structures within the nursing profession. The participants reported that establishing a stronger political presence in the health system is highly relevant for the nursing profession.

In Brazil, nurses are already considered politically active due to the presence of various organisations that advocate for their rights, promote professionalisation, and help influence health policy. Conversely, in Germany, nurses' political engagement is perceived as being nearly absent in debates about development. This is not at least because PHC is seen—and framed in legislation—as the physicians' domain. Until now, the nursing profession has frequently been marginalised from political discourse in Germany. In this context, the further establishment of nursing boards, among other tools, that enable nurses to actively engage in the political sphere, is seen as a crucial driver for advancing APN implementation.

How should there be a change at the level of the legal basis, if there is not enough pressure? (…) That can only be done by organizing nursing boards (…) beginning political lobbying work. (Ger, GP)

At the same time, the nursing participants reported ambivalence within the nursing community itself toward the idea of introducing APN. ‘Some nurses, in the process, also put forth resistance to this; it is not just the physicians (…)’ (Bra, SHO). Concerns are much stronger in Germany, where participants discussed the considerable resistance from practicing nurses to organise themselves into nursing boards.

At the practical level, most of the participants, regardless of country, agreed that at the practitioner level, nurses and physicians ‘have to sit down at the same table’ (Ger, RN) to discuss the division of tasks: ‘What can be delegated? What will be taken over?’ (Ger, RN). From the point of view of a participant, the joint *‘consensus’* (Ger, SHP) to ‘tackle these goals together’ (Ger, SHP) could contribute to pushing for a decision to be made at the macro level.

The perspective of the participating physicians (practitioners and stakeholders) concerning the approval of APN was rather positive. However, an ambivalent willingness among physicians in both countries was stated, perceiving ‘*a profound fear of general practitioners’* (Ger, SHP).

There will certainly be tensions on this issue, especially within the medical profession; there is no doubt about it, that the medical corporation will certainly view this with great suspicion, to say the least. (Bra, SHP)

In addition to physicians with a more negative attitude, participants in both Germany and Brazil also noted that some physicians are highly committed to the approach, encouraging collaboration and showing strong interest in expanding their nursing skills. This aspect, however, was particularly more expressed in the Brazilian groups.

This process will not be in place overnight, and there will be problems in its implementation and in its development, but people need to understand that in the future, this advanced work will improve the population's health as also reduce expenses (…). (Bra, GP)

Finally, experts from both countries recognised the importance of involving the population—and ultimately the patients—as additional dialogue partners in the development of APN roles. They reported that in the current situation, many patients prefer to be treated by physicians, as they are perceived as superior professionals, and patients tend to trust physicians more than nurses.

We really suffer a lot of pressure when we actually take and apply the nursing appointment to the population; they question that they really want to be assisted by a physician and not by a nurse. (Bra, SHO)

In this context, participants, particularly physicians, emphasised the need for greater clarification of the APN concept for the wider population, especially when confusion among patients is noted. They suggested that such clarification could lead to increasing the level of patients' acceptance of nurses assuming advanced practice roles alongside physicians in PHC. In the Brazilian focus groups, however, this was noted with more emphasis by physicians. The participating nurses countered that patients would easily understand this new role since the focus is on care and that users should even be involved in the political process of formulating the APN.

(…) None of my patients use this term, even though I always introduce myself as an APN. (Ger, RN)

The three categories capturing key enablers of and barriers to APN implementation in PHC at different system levels in Germany and Brazil are summarised in Table 4.

## 6 Discussion

This study aimed to identify enablers of and barriers to implementing APN in PHC in Germany and Brazil, focusing on (1) the understanding of the APN role in PHC, (2) its emergence on the political agenda and (3) stakeholder dialogue. Our findings provide new insights into the contextual dynamics of APN implementation, highlighting long‐standing interprofessional hierarchies, the limited political representation of nurses, and a lack of consensus on APN competencies.

### 6.1 APN: An Acronym That Causes Confusion

Our participants perceived limited clarity regarding the term ‘APN’ and their roles as major barriers to APN implementation in PHC. This perception aligns with literature that highlights a global lack of understanding and inconsistent integration of APN roles across different countries, particularly in PHC (Poghosyan et al. 2022). Dowling et al. (2024) explained that APN roles vary worldwide due to the differing stages of development in the nursing profession. Further confusion arises from additional established terms such as ‘advanced nursing practice’ and ‘extended practice’, which contribute to misunderstandings among nurses, physicians, health care professionals and policymakers (Stasa et al. 2014).

In line with this reality, the participants in our study primarily associated advanced practice with clinical roles, whereas other role components—such as leadership, research and management, as outlined by the ICN (2020)—were hardly mentioned. This focus has been described in other studies showing a tendency among practitioners to emphasise clinical aspects of APN while paying less attention to other domains (Schwingrouber et al. 2023). This narrow understanding might contribute to the ongoing confusion about the exact scope of practice for APNs, as evidenced by Torrens et al. (2020), who reported disagreement regarding APN tasks and autonomy levels. This has led participants from both countries to question which tasks should be assigned to APNs, which tasks should remain the responsibility of physicians, and which tasks should become the domain of nurses. The uncertainty around the APN concept can be considered a central barrier to effective APN implementation. In light of this, our findings underscore the need to raise awareness about defining the APN role—in addition to general nurses—to ensure the better utilisation of their capabilities. The results of both the current study and various previous studies suggest that policymakers should prioritise defining APN roles and their scope of practice in their respective health systems (e.g., De Raeve et al. 2024).

In this context, De Raeve et al. (2024) point to the potential value of a unified European regulatory framework and standardised training requirements for fostering greater consistency in APN development. Our study, however, indicates that APN roles inevitably differ due to the complexity and unique characteristics of each health system (ICN 2020); thus, they need to be discussed in national self‐regulation bodies.

### 6.2 Nurses Performing Expanded Practices Already on Track

New nursing tasks in Germany, particularly in PHC, are an increasingly debated issue. Preliminary interim study results report positive experiences regarding patient satisfaction and medication management, which largely correspond to the statements of our participants (Stemmer et al. 2023).

In Germany, the first political initiatives to strengthen advanced nursing competencies are emerging. As the most recent development, in the coalition agreement, the federal government planned the legal establishment of APN roles (CDU et al. 2025).

In Brazil, both research participants and the literature confirm that nurses are already performing advanced tasks such as prescribing medications for people with diabetes or hypertension on the basis of specific nursing protocols established by municipalities varying from region to region (Magnago and Pierantoni 2021). Cassiani and Dias (2022) stressed that neither undergraduate courses nor professional experience should be mistakenly considered sufficient to qualify graduates as APNs. Instead, the importance of enhancing nurse education and restructuring curricula to improve decision‐making skills and critical thinking has been emphasised (Cassiani and Dias 2022). Recently, in Brazil, a national regulatory framework was approved to support APN training through a residency‐integrated master's course (Ministry of Education 2024).

With respect to the expansion of tasks, Germany and Brazil start from markedly different, almost opposite points. In Germany, nurses are still rare in PHC, whereas in Brazil, they are an integral part of the PHC workforce. The differences in the roles and integration of nurses in PHC between the two countries are substantial. In this light, this study highlights that the meaning of ‘advanced’ practice is highly context dependent. For example, tasks such as performing ECGs or drawing blood, which were described as advanced by German participants, are regarded as routine nursing activities in other countries, such as Brazil.

### 6.3 Importance of Dialogue

In our study, fostering dialogue between different stakeholders at the policy and practice level is identified as a crucial enabler of APN implementation. Dialogue is considered essential for crafting inclusive and effective APN development by incorporating diverse perspectives, fostering trust and consensus among stakeholders, and enhancing the relevance and practicality of policy measures (similar for Latin America: Cassiani and Dias 2022). For example, Dovlo et al. (2016) demonstrated how multistakeholder engagement can lead to significant improvements in policy planning and implementation by addressing specific local needs and fostering collaboration among health sector actors.

For Brazil, Miranda Neto et al. (2018) noted that discussions on APN are already emerging ‘among societies, class entities and government agencies aimed to elaborate strategies to nationally implement an expanded model of practice, especially regarding primary health care’. In alignment with our findings, the authors view these discussions as beneficial for formulating strategies for the nationwide implementation of expanded practice models (Miranda Neto et al. 2018).

Compared with German participants, Brazilian participants frequently identified health system managers as important dialogue partners in the APN implementation process. This observation is corroborated by findings from a French study, which emphasised the critical role of collaboration between managers, APNs, and other influential stakeholders. Schwingrouber et al. (2023) highlighted that such collaboration is essential for developing policies and practices that effectively support the recruitment, retention and optimisation of APN roles. This approach facilitates a comprehensive implementation strategy, thus addressing the multifaceted challenges associated with integrating APNs into health systems and ensuring their sustainable contribution to patient care (Schwingrouber et al. 2023).

Dialogue should take place not only at the political level but also at the practical level. The exchange between physicians and nurses, which is reported to be welcome by both parties, is seen as crucial for advancing the implementation of the APN. Interestingly, the participating physicians reported being open to and, in some cases, even supportive of this implementation. This is particularly noteworthy because practicing nurses reported a more nuanced or even rejecting attitude towards APN implementation from the physicians with whom they interact in their daily practice. The ambivalent attitudes of doctors observed herein are similarly reflected in the literature (Magnago and Pierantoni 2021).

Our findings can be meaningfully interpreted through the lens of Habermas' theory of communicative action (Habermas 1981). Habermas (1981) emphasises rational discourse, a form of communication in which participants engage openly, equally, and without coercion, with the aim of reaching a shared understanding through argument‐based reasoning. In the context of APN implementation, this would require communicative spaces resembling an ideal speech situation, enabling all stakeholders—including nurses—to participate on equal terms and without structural distortion. In practice, however, as our findings show, implementation unfolds within real communication settings shaped by power imbalances and institutional barriers—asymmetries that are particularly evident in Germany (see also Franco et al. 2025). Thus, for sustainable APN implementation, inclusive communicative structures—in the sense of genuine dialogue—are not only practically necessary but also—from a theoretical perspective—essential for approximating an ‘ideal speech situation’ (Habermas 1981).

### 6.4 Nurses' Engagement in the Political Arena

Our findings highlight the critical importance of political nursing activity as a key enabler for the implementation of APN in PHC. According to Unsworth et al. (2022), the successful establishment of APN roles requires robust support from nurses at the national level. In many countries, the lack of nurses in government positions or the presence of nurses without sufficient influence has frequently impeded national discussions on APN roles (Unsworth et al. 2022). Therefore, the involvement of nurses in national‐level discussions and policy‐making is essential for the effective implementation of the APN role.

In Brazil, as indicated by the participants and corroborated by the literature, ABEn and COFEN are recognised as two pivotal organisations that advocate for the interests of nurses at the political level (Bula et al. 2024). Both organisations have significantly contributed to bringing the expansion of APNs onto the political agenda (Minosso and Toso 2021). Despite the relatively high level of nurses' political activity in comparison with that in Germany, studies suggest that there is nevertheless a pressing need for increased political engagement and mobilisation among nurses in Brazil (Figueira et al. 2020). In contrast, German participants lament the low level of organisation within the nursing profession, which hinders effective political influence. This situation results in the nursing profession in Germany continuing to be subject to external control and lacking a national representation, thereby limiting its political say; this is a topic that was heavily discussed in the focus groups of the current study, particularly in reference to the absence of a national nursing board. Consequently, although the ongoing political engagement of nurses in Brazil is often perceived as insufficient, the German nursing profession could still learn from the Brazilian example. Nevertheless, recent policy developments indicate a potential shift: the coalition agreement of the new federal government outlines the intention to enhance nurses' voices in health care governance (CDU et al. 2025).

### 6.5 Nurses Should Be More Involved

In their review, Torrens et al. (2020) reported that a primary factor hindering APN implementation is a lack of confidence in nurses' competence or ability to perform the role, with self‐doubt affecting their ability to prescribe, make autonomous decisions or assume leadership. Researchers have further emphasised that professional self‐confidence is crucial for the successful execution of clinical skills and effective relationships with patients and colleagues (Makarem et al. 2019). The lack of self‐confidence was reported to be a prominent inhibiting factor by the German focus groups. Another uncertainty stems from the concern among some participating nurses from both countries that the expansion of APN is intended primarily to fill gaps in the workforce rather than elevate their professional status. This perspective corresponds to the historical reasons for implementing APNs to address shortages in underserved regions (Delamaire and Lafortune 2010), which is, however, not seen as a prudent pathway to APN implementation by our participants.

Based on these observations, stronger emphasis should be placed on understanding the potential fears, desires and perceptions of nurses concerning the expansion of their roles. Incorporating nurses and considering their needs in the development process could not only prove beneficial but also demonstrate appreciation for the nursing profession.

### 6.6 Regulation and Lack of a Legal Framework: Well‐Known Barriers

Finally, our study confirms the importance of providing a legal framework that enables APN at the national level. Such a framework needs to legitimise and develop APN through the multiple roles of APNs, including prescribing, leadership, management and education. Notably, the ability to conduct safe medication reviews is usually dependent on the ability and authority to prescribe medicines. Brownwood and Lafortune (2024) showed that countries that have successfully established APN roles did so based on robust legislation and regulations that define the scope of practice for APNs. Such changes could also help clarify funding options, which are a significant concern for our participants and a topic that is widely discussed in the literature (Torrens et al. 2020). However, to achieve these changes in the legislative framework, a ‘mind shift’ among policymakers is crucial, as Schober et al. (2016) suggested, and is indispensable for successful implementation.

### 6.7 The PEPPA Framework as Implementation Model: Analytical Value and Contextual Limitations

The enablers of and barriers to implementing APN roles in Germany and Brazil identified in this study correspond closely with the stages of the PEPPA framework (Bryant‐Lukosius and Dicenso 2004). The results specifically reflect the framework's emphasis on defining roles, fostering stakeholder engagement, and establishing legal and financial support for APN in PHC. A key finding of this study is the ambiguity surrounding the term ‘APN’, which underscores the need for clear role definitions as prescribed in step 5 of the PEPPA framework.

Stakeholder engagement, or the ‘dialogue’ emphasised in the PEPPA framework, is seen herein as essential for integrating APN into national health care agendas, with participants calling for collaborative engagement across nurses, physicians, policymakers and the public to develop a shared understanding of APN roles. Although participants reported supporting APN as a means by which to strengthen PHC and broaden patient access, it is noteworthy that patient needs—which are central to the PEPPA framework—were not a focal point emphasised in these discussions; this reflects criticisms present in the literature on implementing new care models without having sufficient patient input (Schaeffer and Hämel 2020). While the PEPPA framework offers a robust outline for APN role development, it falls short in addressing the sociopolitical complexities specific to regions such as Germany and Brazil, where rigid regulations and liability concerns persist. Additionally, it assumes a supportive regulatory environment, which is not always present in these settings.

### 6.8 Strengths and Limitations

To the best of our knowledge, this study represents the first scientific investigation on the barriers to and enablers of APN implementation in PHC in both Germany and Brazil. One strength of this study is the inclusion of insights from stakeholders at the federal and regional political levels, as well as from experts at the practitioner level, thus facilitating a comprehensive, multiperspective analysis. Another key strength is the use of a binational research team, which brings together professionals from different fields, particularly nurses and physicians.

However, this study also has several limitations. The multiple languages and cultures involved in this research required intensive communication and attention between the researchers to fully understand the contexts from a comparative perspective. In addition, the sampling included participants who seemed to be very supportive or at least open to APN implementation in PHC, which may have introduced response bias. Importantly, however, the research team placed significant emphasis on achieving balanced representation during recruitment.

## 7 Conclusion

In light of increasingly complex global health needs, the development of APN roles in PHC has gained support within the international health agenda, as advocated by the WHO. Our study highlights that the process of implementing APNs relies on collaboration among policymakers, professional bodies, and practitioners at the national, regional and local levels. While there is significant potential for APNs to enhance PHC in Germany and Brazil, implementing this model is complex and demands a multifaceted approach. To move forward, it is essential for both countries to address professional and systemic challenges in ways that reflect their unique contexts. In addition, prioritising the clarification of the APN concept and advancing its regulation on the political agenda will be key to achieving progress. Moreover, for both countries, efforts are needed to regulate APN autonomy and fair compensation to fully integrate APNs and address health care hierarchies. Ongoing research should focus on increasing the level of public awareness as the APN field advances, thereby contributing valuable insights to informing policies on APN development.

## Author Contributions

D.B.: Conceptualisation of the overall research, conceptualisation of the study, data collection, formal analysis, interpretation, writing of the first draft, writing of the manuscript, writing review and editing. C.M.F.: Conceptualisation of the study, formal analysis, interpretation, writing the manuscript, writing the review and editing. M.H.: Conceptualisation of the overall research, conceptualisation of the study, data collection, formal analysis, writing the review and editing. L.G.: Data collection, writing the review and editing. B.R.G.O.T.: Conceptualisation of the overall research, conceptualisation of the study, data collection, interpretation, writing the review and editing, supervision. K.H.: Conceptualisation of the overall research, conceptualisation of the study, formal analysis, interpretation, writing the manuscript, writing the review, and editing, supervision.

## Ethics Statement

Ethical approval and permission to conduct the study were obtained from the ethics committees of Bielefeld University (reference number: 2022‐87) and Western Paraná State University (UNIOESTE) (reference number: 5.349.117). Before conducting the focus group interviews, participant information, including the purpose of the study, the course of the focus groups and the data protection measures implemented, was provided. All participants provided written consent to participate in this study. Data confidentiality was strictly guaranteed throughout the entire study based on a contract between the German and Brazilian research institutions. The data were processed solely via the Sciebo website, a noncommercial cloud storage platform used by public research institutions in the German state of North Rhine‐Westphalia and secure network drives owned by Bielefeld University.

## Conflicts of Interest

B.R.G.O.T. is a vice‐president of the APN Network of Latin America and the Caribbean, an ambassador for the American Association of APNs, the president of the Brazilian Association of APN and a member of the Core Steering Group of the International Council of Nurses (ICN) Nurse Practitioner (NP)/Advanced Practice Nurse (APN) Network. All authors declare that they have no competing interests related to this work.

## Data Availability

The data are available upon request due to privacy‐related restrictions. The datasets generated and/or analysed during the current study are available from the corresponding author upon reasonable request.
